# Combination of meshed dermis graft and cultured epithelial autograft for massive burns: Three case reports

**DOI:** 10.1097/MD.0000000000013313

**Published:** 2018-11-30

**Authors:** Minoru Hayashi, Kotaro Yoshitake, Ryohei Tokunaka, Yuki Yoshida, Mikiko Oshima, Sayo Tatsuta, Taishi Hamada, Ayako Kamitomo, Akito Hamajima

**Affiliations:** a Department of Plastic, Reconstructive and Aesthetic Surgery, Japan Red Cross Maebashi Hospital; b Department of Plastic Surgery, Gunma Children's Medical Center, Gunma, Japan.

**Keywords:** artificial dermis, Burns, CEA, cultured epithelial autografts, dermis graft, transmission electron microscopy

## Abstract

**Rationale:**

This study reviewed the use of a combination of meshed dermis graft and cultured epithelial autografts (CEA) made in Japan “JACE” (JACE; Japan Tissue Engineering Co., Ltd. Japan) for the treatment of massively burns. JACE is a Green-type CEA. We recently described a method in which we prepare the wound bed for burned patients by using artificial dermis and graft with JACE on a meshed 6:1 split-thickness autograft. In this report, we used a meshed 3:1 split-thickness dermis graft without epithelial cells. There are several reports of combination of using CEA on meshed split-thickness autograft, however this is the first report of using CEA on meshed split-thickness dermis graft.

**Patient concerns and diagnosis:**

Between March 2015 and August 2017, 3 burn patients were enrolled in this study. The patients ranged in age from 51 to 66 years. All 3 patients suffered severe burn injury that caused by flame. % Total Body Surface Area (TBSA) burned were ranged from 37.5% to 69%.

**Interventions:**

All patients received surgical treatment with tangential excision within a week from admission. We implanted artificial dermis immediately after debridement. Basically, we applied meshed 6:1 split-thickness autografts to the wound bed and covered with JACE. However, in the absence of split-thickness autografts, we used a meshed 3:1 split-thickness dermis graft instead of a meshed 6:1 split-thickness autograft.

**Outcomes:**

At 3 weeks after the transplantation of JACE, the take rate for JACE sheets was >60% on the meshed 3:1 split-thickness dermis graft. Furthermore, almost all of the burn wounds had healed at 6 weeks after surgery.

**Lessons:**

We observed good results by grafting JACE on meshed 3:1 dermis graft. With this new method, it is possible to cover a large burn wound by harvesting tissue from only a small site.

## 1 Introduction

Severely burned patients can require many clinical treatments including burn resuscitation, respiratory support, nutritional support, infection control, pain management, dressing and surgery. Advances in medical treatments have helped to improve survival rates.^[1,2]^ Cultured epithelial autografts (CEA) were used clinically for the first time in the early 1980s. Since 1984, when good results were achieved in extensively burned children through the use of CEA, the usefulness of Green-type CEA has become widely recognized.^[3–5]^ CEA made in Japan “JACE” (JACE; Japan Tissue Engineering Co., Ltd. Japan) is a Green-type CEA that was developed in Japan. Since 2009, JACE has often been used for >30% Total Body Surface Area (TBSA) burn patients.

In a previous report, we prepared the wound bed for burned patients by using artificial dermis and graft with JACE on a meshed 6:1 split-thickness autograft.^[6]^ We obtained good results and observed the engraftment process by both transmission electron microscopy and scanning electron microscopy. We reported that the dermis from the autograft extended underneath JACE, and there was binding between JACE and the dermis.^[7]^

In the present study, we treated 3 massively burned patients. We used JACE on a meshed 3:1 split-thickness dermis graft instead of a meshed 6:1 split-thickness autograft for part of the burn wound. We achieved good results in all 3 cases.

This report describes the use of a combination of meshed dermis graft and JACE for the treatment of massively burned patients. There are several reports of the combination of using CEA on meshed split-thickness autograft; however, this is the first report of using CEA on meshed split-thickness dermis graft.

## 2 Methods

Burn patients who were treated with a combination of meshed dermis graft and CEA were reviewed. Between March 2015 and August 2017, 3 burn patients were enrolled in this study, which was approved by the institutional review board (Table 1). The patients ranged in age from 51 to 66 years at the time of admission. All 3 patients were male and % (TBSA) burned were ranged from 37.5% to 69%.

**Table 1:**
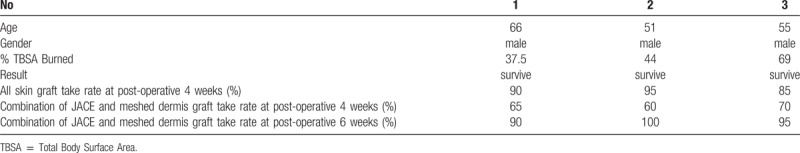
Patients details.

JACE was ordered from Japan Tissue Engineering Co., Ltd. (Aichi, Japan) after we obtained consent from the patients’ families. We harvested full-thickness normal skin from unburned areas within 48 hours. We were able to use JACE 3 weeks after this harvest.

All patients received surgical treatment with tangential excision within a week from admission, and all necrotic tissue was removed. We implanted artificial dermis immediately after debridement. A week later, we began to apply dressings using Vaseline petroleum jelly and Trafermin (Fiblast, Kaken Pharmaceutical Co., Ltd., Japan) every day to construct a wound bed.^[8,9]^

Basically, we applied meshed 6:1 split-thickness autografts to the recipient wound bed and covered with JACE. However, in the absence of split-thickness autografts, because of extensive burn and lack of intact skin area, we used meshed 3:1 split-thickness dermis graft instead of meshed 6:1 split-thickness autograft. After implanting JACE, we followed Sood protocol for cultured epithelial autograft dressings.^[10]^ In the immediate postoperative period, the grafted areas were left exposed to air for 4 hours each day to allow the JACE grafts to dry. Takedown (removal of the silicone gauze from the JACE graft) generally occurred 1 week after placement of the grafts. Dressings were applied with Vaseline petroleum jelly after takedown. Almost all of the wounds closed within 4 to 6 weeks.

## 3 Case reports

### 3.1 Case 1

A 69-year-old Asian male was suffered severe burn injury that caused by flame. The patient had no known comorbidity but had drunk much alcohol every day. The patient has second to third-degree burns on face, chest, abdomen, both arms, and both buttocks that affected 37.5% TBSA. Second-degree burn was estimated 9.5% TBSA and third-degree burn was estimated 28% so that Burn Index was 32.75. Upon admission and after cleaning the fresh burn and removing blisters, we changed dressing every day. We harvested full-thickness normal skin from right groin area in order to manufacture JACE on next day. We performed debridement all eschar on 7 days from admission. And we implanted artificial dermis on the all ulcer to manage the good wound bed. At last, we applied JACE on meshed 3:1 split-thickness dermis graft or meshed 6:1 split-thickness autograft for covering all wound. All skin graft take rate was 90% at four post-operative weeks. And this patient was transferred to a rehabilitation hospital on 101 days from admission.

### 3.2 Case 2

A 51-year-old Asian male was suffered severe burn injury with inhalation injury that caused by flame. The patient had no known comorbidity. The patient has second to third-degree burns on face, chest, abdomen, right side of the back, both arms, right thigh and left lower leg that affected 44.0% TBSA. Second-degree burn was estimated 34% TBSA and third-degree burn was estimated 10% so that Burn Index was 27. We harvested full-thickness normal skin from right groin area in order to manufacture JACE on 2 days from admission. And we performed debridement all eschar and we implanted artificial dermis on the all ulcer on the same day. We implanted meshed split thickness skin graft on back in advance on 17 days from admission. This is because the patient will need absolute rest after using JACE and generally CEA take rate on back is very low. We finally applied JACE on meshed 3:1 split-thickness dermis graft or meshed 6:1 split-thickness autograft on chest and abdomen and implanted only meshed 3:1 split-thickness autograft for upper and lower limbs wound on 24 days from admission. All skin graft take rate was 95% at four post-operative weeks. And this patient was transferred to a rehabilitation hospital on 77 days from admission.

### 3.3 Case 3

A 55-year-old Asian male was suffered severe burn injury with inhalation injury that caused by flame. The patient had no known comorbidity. The patient has second to third-degree burns on face, chest, abdomen, back, both upper and lower limbs that affected 69.0% TBSA. Second-degree burn was estimated 48% TBSA and third-degree burn was estimated 21% so that Burn Index was 45. Upon admission and after cleaning the fresh burn and removing blisters, an escharotomy was performed. We harvested full-thickness normal skin from right groin area in order to manufacture JACE on the next day from admission. And we performed debridement all eschar and we implanted artificial dermis on the all ulcer on 6 days from admission. We applied JACE on meshed 3:1 split-thickness dermis graft on both upper limb and chest and implanted only meshed 3:1 split-thickness autograft for the rest wound on 27 days from admission. All skin graft take rate was 85% at four post-operative weeks. And this patient was transferred to a rehabilitation hospital on 86 days from admission.

## 4 Discussion

In 1963, Todaro and Green found 3T3 cells from mouse embryo cells.^[11]^ In 1975, Rheinwald and Green described the technique of cultivating autologous keratinocytes for autografting.^[12]^ The CEA was used clinically for the first time in the early 1980s. Since a report described that extensively burned children recovered by using CEA in 1984, the usefulness of Green-typed CEA became known widely.^[3–5]^ Epicel (EPICEL; Vericel Corporation. USA) has been commercialized in United States as the first tissue engineering product in the world since 1988. After 20 years, JACE was developed and was also a Green-type autologous cultured epidermis. However, this was not a late-coming article of EPICEL because the process of manufacture was different.

In Japan, the wound bed is often prepared using artificial dermis due to the scarcity of cryopreserved cadaver allograft.^[6]^ In such cases, collagen is poorly constructed, and the combination of JACE and meshed split-thickness autograft has a better take rate than JACE alone. A 6-year surveillance of the use of JACE in Japan found that the average take rate for graft sites treated with a combination of JACE and meshed autograft was 77 ± 29%.^[13]^

The reason for the better engraftment rate is that dermis extends and connects from the autograft and fills the mesh and strongly binds JACE.^[7]^ In this previous report, we undertook skin biopsies after transplantation of JACE and observed the engraftment process of JACE. We implanted JACE on meshed 6:1 split thickness autografts and we focused on the boundary of the autograft and JACE. In the scanning electron microscope, it was observed that papillary dermis migrated toward the central portion. And this dermis strongly bound JACE. If dermis can extend, we thought that only dermis would engraft. We implanted JACE on a meshed dermis graft and obtained good results.

**Figure 1:**
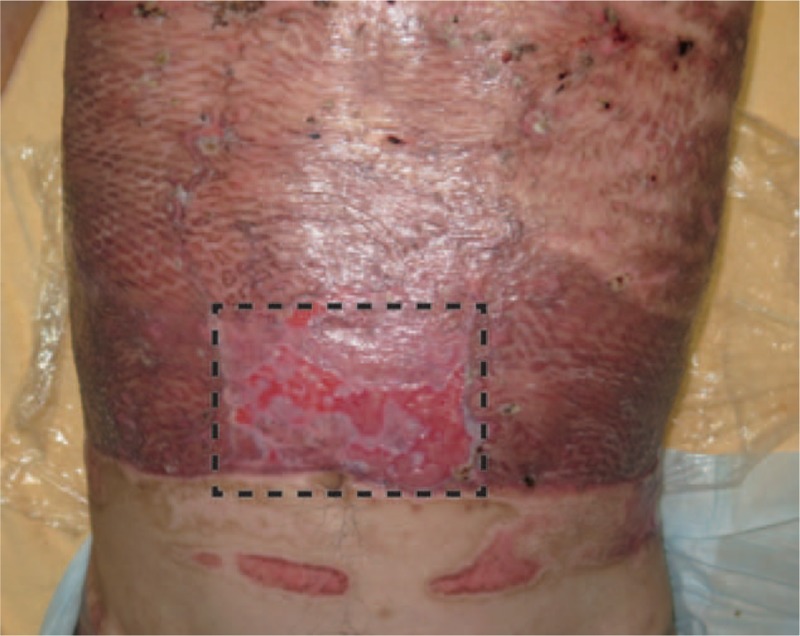
Three weeks after the transplantation of JACE. Inside the black dotted line, we implanted JACE on a meshed 3:1 split-thickness dermis graft. The take rate of the JACE sheets was > 60%. Outside the black dotted line, we implanted JACE on meshed 6:1 split-thickness autograft, as usual. Almost all of the JACE sheets were taken.

**Figure 2:**
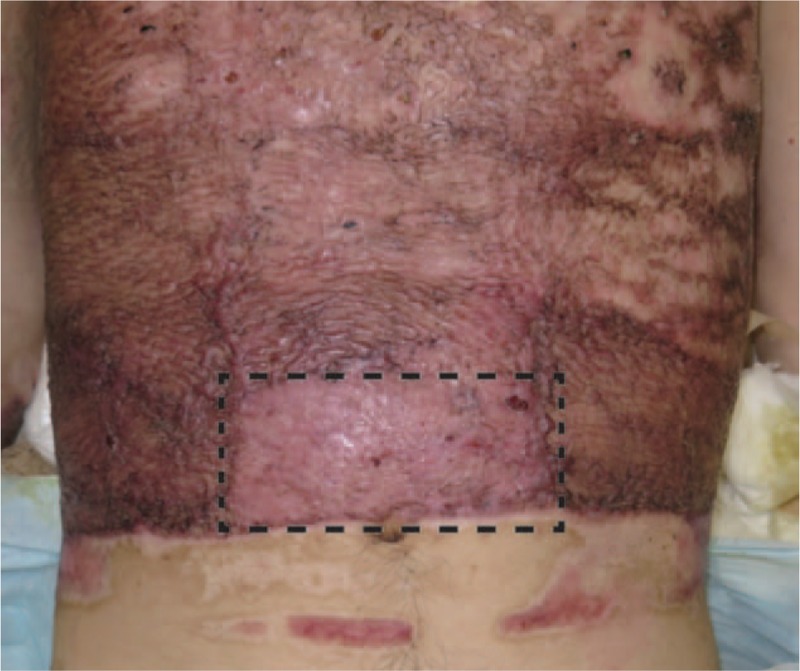
Six weeks after the transplantation of JACE. Inside the black dotted line, we implanted JACE on a meshed 3:1 split-thickness dermis graft. Outside the black dotted line, we implanted JACE on a meshed 6:1 split-thickness autograft. All of the burn wounds had healed.

In this report, three patients were treated with a combination of meshed dermis graft and CEA. The follow-up period was 7 months to 3 years, with a mean of 1.9 years. All three patients were tolerated the procedure well without any complications. We present a case of No 2. Figure 1 shows the results at 3 weeks after the transplantation of JACE. Inside the black dotted line, we implanted JACE on a meshed 3:1 split-thickness dermis graft. The take rate of the JACE sheets was >60%. Outside the black dotted line, we implanted JACE on meshed 6:1 split-thickness autograft, as usual. Almost all of the JACE sheets were taken. Figure 2 shows the results at 6 weeks after surgery. All of the burn wounds had healed.

We obtained skin biopsy samples at three and 6 weeks after surgery and observed both by transmission electron microscopy (Figs. 3 and 4). We recognized the basement membrane, which showed binding between JACE and subcutaneous tissue. The thin lines and dots on the subcutaneous tissue are collagen fibers, which are longer, thicker and at a higher density at 6 weeks compared to 3 weeks. This indicates that JACE has engrafted with the meshed 3:1 split-thickness dermis graft.

**Figure 3:**
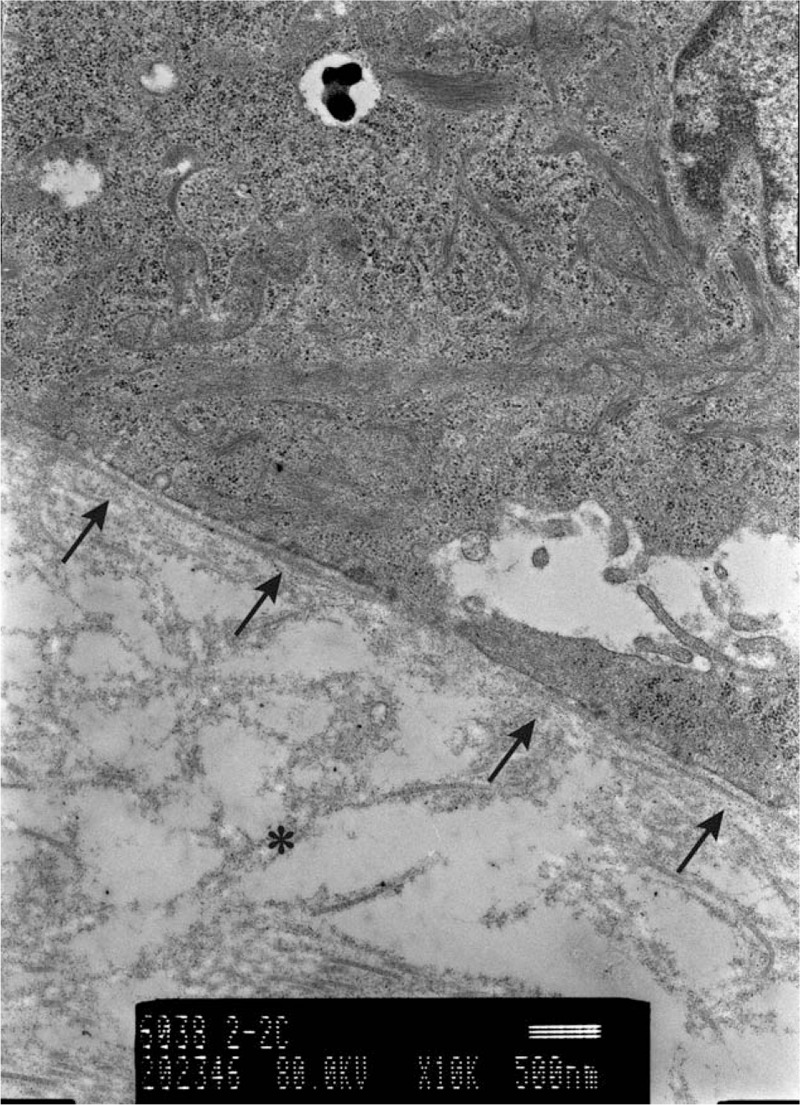
[{(Figs. 3 and 4.)}] We obtained skin biopsies with a combination of meshed dermis graft and JACE at 3 and 6 weeks after surgery. The biopsy site was a gap in the meshed dermis graft. The arrows show the basement membrane. The upper side of the basement membrane contains JACE and the bottom side is subcutaneous tissue. The basement membrane serves to bind JACE and subcutaneous tissue. The asterisk shows the gathering of collagens for subcutaneous tissue by artificial dermis. The thin lines and dots are collagen fibers, which are more longer, thicker and at a higher density at 6 weeks compared to 3weeks. These indicate that JACE has engrafted with the meshed 3:1 split-thickness dermis graft.

**Figure 3 (Continued):**
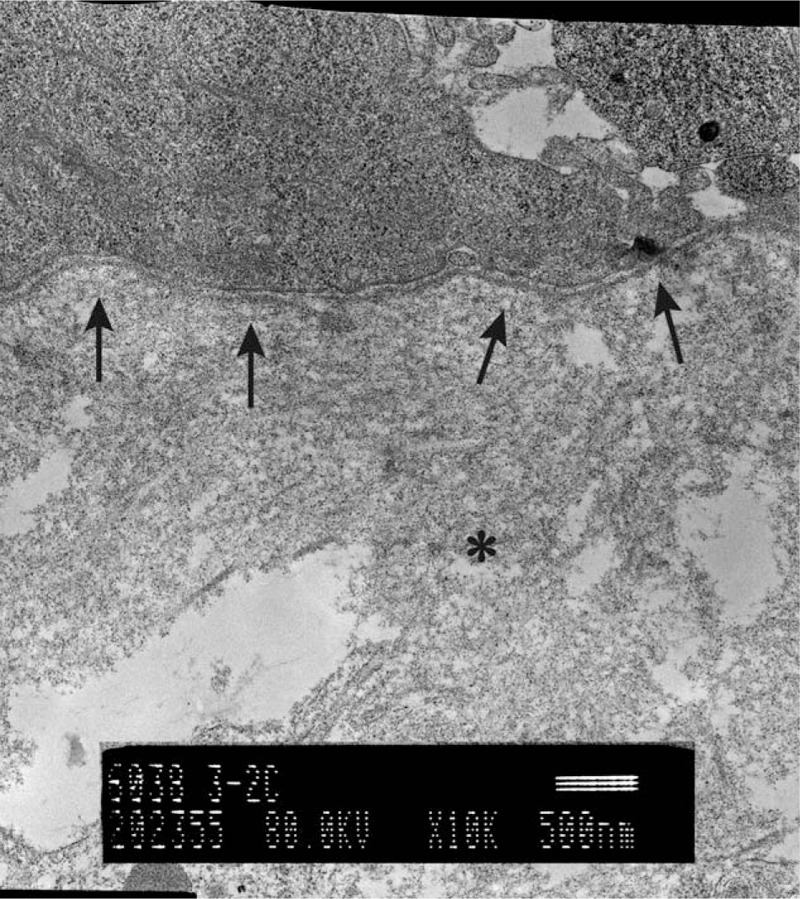
[{(Figs. 3 and 4.)}] We obtained skin biopsies with a combination of meshed dermis graft and JACE at 3 and 6 weeks after surgery. The biopsy site was a gap in the meshed dermis graft. The arrows show the basement membrane. The upper side of the basement membrane contains JACE and the bottom side is subcutaneous tissue. The basement membrane serves to bind JACE and subcutaneous tissue. The asterisk shows the gathering of collagens for subcutaneous tissue by artificial dermis. The thin lines and dots are collagen fibers, which are more longer, thicker and at a higher density at 6 weeks compared to 3weeks. These indicate that JACE has engrafted with the meshed 3:1 split-thickness dermis graft.

With this method, we can harvest multiple times at the same site, and can use JACE on meshed autograft and meshed dermis graft for burn wounds. A meshed dermis graft requires a longer healing period than a meshed split-thickness autograft, and thus, depending on the specific wound, a meshed dermis graft may not be the optimal choice. However, with this method, we can cover a large burn wound by harvesting from only a small site.

This new method may change the strategy for treating massively burned patients.

## 5 Patient consent

All content and procedures described within conform to the principles outlined in the Declaration of Helsinki. All patients or their families provided their written informed consent to participate. The institutional ethics committee approved the study protocol.

## Acknowledgments

The authors thank Dr Hideyuki Muramatsu for technical advices with the treatments. And we also thank all the medical staff for the work and enthusiasm.

## Author contributions

**Validation:** Kotaro Yoshitake, Ryohei Tokunaka, Yuki Yoshida, Mikiko Oshima, Sayo Tatsuta, Taishi Hamada, Ayako Kamitomo, Akito Hamajima.

**Writing – original draft:** Minoru Hayashi.
